# Supplementary material for: The relationship between interpersonal distance preference and estimation accuracy in autism
Source: PLoS One. 2024 Sep 9;19(9):e0306536. doi: 10.1371/journal.pone.0306536 (PMC11383220; doi:10.1371/journal.pone.0306536)
Supplement: S1 File — (PDF) [file pone.0306536.s001.pdf]

In the ASD group, IQ was assessed using the Test of Nonverbal Intelligence (TONI-4; Brown et al., 2010), with an average MIQ of 102.51 (SDIQ = 12.65, range: 84-122). Educational attainment within the ASD group included completion of high school by all participants, with 34.6% holding a bachelor's degree and 3.8% holding a master's degree. Regarding comorbidity, 23% reported a diagnosis of Attention Deficit Disorder (ADD) or Attention Deficit Hyperactivity Disorder (ADHD), 11% reported Obsessive-Compulsive Disorder (OCD), and 3% reported borderline personality disorder. Additionally, 48% reported no comorbidity, while the rest preferred not to disclose this information. Concerning medications, 50% reported receiving medication for ADHD, a mood disorder, or both, while 38% were not medicated, and 22% preferred not to disclose this information.
